# Severe Lower Urinary Tract Dysfunction in Otherwise Healthy Children: A Three-Case Series and Narrative Review

**DOI:** 10.3390/pediatric18010020

**Published:** 2026-02-03

**Authors:** Olivia-Oana Stanciu, Andreea Moga, Laura Balanescu, Mircea Andriescu, Ruxandra Caragata, Radu Balanescu

**Affiliations:** 1 Department of Pediatric Surgery and Orthopedics, “Carol Davila” University of Medicine and Pharmacy, 050474 Bucharest, Romania; olivia.stanciu@drd.umfcd.ro (O.-O.S.); laura.balanescu@umfcd.ro (L.B.); mircea.andriescu@umfcd.ro (M.A.); ruxandra.caragata@umfcd.ro (R.C.); radu.balanescu@umfcd.ro (R.B.); 2 Pediatric Surgery Department, “Grigore Alexandrescu” Clinical Emergency Hospital for Children, 011743 Bucharest, Romania

**Keywords:** lower urinary tract dysfunction, non-neurogenic bladder, Hinman syndrome, pediatric urology, urodynamics, case series

## Abstract

Background: Severe lower urinary tract dysfunction (LUTD) in neurologically and anatomically normal children is uncommon and frequently underdiagnosed. When severe, functional voiding disorders may closely mimic obstructive or reflux pathology, leading to diagnostic errors, unnecessary invasive procedures, and potential risk to the upper urinary tract. Case presentation: We present three pediatric cases (aged 3–10 years) referred for recurrent febrile urinary tract infections, incontinence, or acute urinary retention in the absence of neurological or structural abnormalities. Urodynamic evaluation identified three distinct severe functional phenotypes: detrusor overactivity with reduced bladder capacity, poor compliance with detrusor–sphincter dyssynergia and secondary high-grade vesicoureteral reflux (Hinman syndrome), and detrusor underactivity with significant post-void residual volumes. All patients demonstrated marked bladder wall remodeling on cystoscopy, including trabeculation and pseudopolypoid mucosal changes. Case discussion: Despite similar clinical severity, the cases illustrated substantial functional heterogeneity and differing risks of upper urinary tract involvement. Urodynamic phenotyping proved central to diagnosis, differentiation from structural disease, and treatment planning. Multimodal conservative management—including urotherapy, pelvic floor biofeedback, targeted pharmacologic therapy, and, when indicated, clean intermittent catheterization or antibiotic prophylaxis—led to resolution of recurrent infections and meaningful improvement in bladder function during medium-term follow-up, although symptom recurrence occurred in one patient after treatment withdrawal. Conclusions: These cases highlight the heterogeneity and potential reversibility of severe functional LUTD in otherwise healthy children. Early functional recognition based on urodynamic assessment is essential to avoid misdiagnosis, prevent unnecessary surgical intervention, and protect renal function. Conservative, function-oriented management remains the cornerstone of effective treatment. The findings are discussed in the context of the existing literature on severe non-neurogenic LUTD and Hinman syndrome.

## 1. Introduction

Lower urinary tract dysfunction (LUTD) encompasses a heterogeneous spectrum of disorders characterized by abnormalities of bladder storage or voiding [1]. While mild or moderate LUTD—such as overactive bladder, dysfunctional voiding, or mild post-void residuals—is relatively common in pediatric populations, severe presentations involving persistently high post-void residual volumes, episodic or chronic urinary retention, intractable incontinence, or secondary upper urinary tract changes remain rare among neurologically intact children [2,3].

Traditionally, severe LUTD in children has been considered a marker of underlying neurogenic bladder, often associated with identifiable structural or developmental anomalies such as spinal dysraphism, myelomeningocele, tethered cord, sacral agenesis, or occult spinal lesions [3,4]. However, an emerging body of literature suggests that a subset of otherwise healthy children—i.e., with no detectable neuroanatomical, systemic, or congenital urinary tract defects—may present with severe lower urinary tract dysfunction [5,6]. The pathophysiology in these cases remains poorly understood and is likely multifactorial. Proposed mechanisms include maladaptive or learned voiding behaviors (such as chronic withholding), psychogenic and psychological stressors, subtle functional immaturities or dysregulation of bladder sensory/afferent pathways, and autonomic dysregulation of detrusor contractility [5,7].

An additional and clinically significant feature in many of these cases is the occurrence of recurrent urinary tract infections (rUTIs). Recurrent UTIs not only reflect impaired bladder emptying and stasis but also pose risks of renal scarring, hypertension, and long-term renal damage, especially in children with underlying voiding dysfunction [8,9]. Indeed, persistent residual urine, incontinence, high bladder pressures, and dysfunctional voiding patterns are recognized risk factors for bacterial colonization and recurrent infections [8,10]. Thus, the coexistence of severe LUTD and rUTIs in children without overt anatomical or neurological abnormalities presents both a diagnostic puzzle and a therapeutic challenge.

In this manuscript, we describe three paediatric cases of severe lower urinary tract dysfunction (LUTD) occurring in otherwise healthy children who presented with recurrent urinary tract infections (UTIs) but had no identifiable neurological or structural urological abnormalities. We also review the current literature to contextualize these findings within the broader spectrum of functional and non-neurogenic lower urinary tract dysfunction in children. In accordance with ICCS recommendations, severe LUTD in this report refers to functionally significant voiding disorders characterized by sustained high detrusor pressures, poor compliance, marked post-void residuals, and/or recurrent febrile UTIs, representing the severe end of the non-neurogenic LUTD spectrum [11].

The three cases were deliberately selected to illustrate distinct and severe phenotypes within the non-neurogenic LUTD spectrum—detrusor overactivity, detrusor–sphincter dyssynergia (Hinman syndrome), and detrusor underactivity—each associated with significant morbidity and risk of misdiagnosis. Together, they illustrate how severe functional LUTD can mimic obstructive or reflux pathology, highlight common diagnostic pitfalls, and emphasize the potential for functional recovery when appropriate conservative management is instituted. Accordingly, this manuscript aims to illustrate diagnostic reasoning and management principles rather than to quantify disease frequency or outcomes.

## 2. Case Series

### 2.1. Methods

This work represents a retrospective descriptive case series of three pediatric patients evaluated between January 2018 and December 2024 during routine tertiary-care practice. The cases were identified during routine clinical practice and purposively selected for detailed presentation because they illustrated distinct urodynamic phenotypes of severe lower urinary tract dysfunction (LUTD) in neurologically and anatomically normal children. This work is purely illustrative and descriptive, not derived from a screened cohort. This case series is reported with reference to the CARE guidelines for case reports, adapted to a descriptive, illustrative case series format, with emphasis on clinical transparency and outcome reporting rather than population inference.

Each child presented with recurrent urinary tract infections (rUTIs) and severe functional voiding abnormalities confirmed by imaging and urodynamic studies. All underwent comprehensive clinical, radiologic, and endoscopic evaluation to exclude neurogenic or structural causes. Neurological integrity was verified by complete neuro-urological examination. Neurological evaluation included detailed clinical assessment of lumbosacral reflexes, gait, anal tone, and perineal sensation. Electromyography (EMG) and spinal magnetic resonance imaging (MRI) were not performed, as none of the patients displayed neurological symptoms or examination findings suggestive of spinal or peripheral nerve pathology. Therefore, the diagnosis of non-neurogenic LUTD was based on clinical exclusion of neurological disease.

These three cases were encountered over a six-year period (2018–2024) within our tertiary pediatric urology practice. They were selected for detailed illustration because they typify distinct severe functional LUTD phenotypes (overactive, dyssynergy, and underactive). Other patients with milder or incomplete forms of LUTD were not included, as the present report focuses on the most severe, diagnostically challenging presentations.

Because this is a case series rather than a screened cohort, no formal recruitment flow chart or denominator population was applied. Nevertheless, all three patients shared several clinical characteristics relevant to severe functional LUTD, summarized to clarify the clinical context rather than to imply systematic screening (Table 1): neurologically and anatomically normal on examination and imaging; recurrent febrile or culture-proven UTIs; urodynamic evidence of detrusor overactivity, dyssynergia, or underactivity consistent with severe LUTD; no prior lower urinary tract surgery related to functional obstruction at the time of initial LUTD evaluation, and no systemic disease influencing voiding. One patient (Case 2) had previously undergone ureteral reimplantation for grade V vesicoureteral reflux before referral. Because she later illustrated persistent LUTD despite anatomically successful surgery, she was included as a paradigmatic case illustrating diagnostic and therapeutic pitfalls, rather than as part of the initial eligibility framework. Case 2 was intentionally retained as a paradigmatic example of misclassification, illustrating how severe functional LUTD may persist despite anatomically successful surgery when the underlying voiding dysfunction is not recognized.

For the purpose of this series, severe lower urinary tract dysfunction (LUTD) was defined by the presence of one or more of the following objective or clinical criteria: (1) urodynamic thresholds indicating marked functional impairment—detrusor pressure exceeding 40 cm H_2_O during filling or voiding, bladder compliance < 10 mL/cm H_2_O, or post-void residual volume > 20% of the expected bladder capacity for age on at least two evaluations; (2) symptom persistence or recurrence despite ≥3 months of standard urotherapy (timed voiding, hydration, bowel management); (3) refractory or recurrent infection profile, defined as ≥2 febrile UTIs or ≥3 culture-proven UTIs within 12 months despite appropriate treatment. These criteria were chosen to reflect clinically significant functional compromise warranting tertiary evaluation and multimodal therapy. These thresholds are not intended as universal diagnostic cutoffs but were selected based on commonly cited risk markers for bladder decompensation, infection recurrence, and upper tract involvement reported in the pediatric urodynamic literature. We acknowledge that international guidelines (including ICCS) emphasize pattern recognition rather than strict numerical thresholds, and that definitions of “severity” may vary across studies [1,2].

Clinical data extracted from medical records included demographic information, presentation, imaging and urodynamic findings, cystoscopic appearance, management strategy, and outcomes during 18–36 months of follow-up (median = 28 months). Follow-up duration was 30 months (Case 1), 36 months (Case 2), and 24 months (Case 3). Patients were reviewed at 3, 6, and 12 months after diagnosis and at least annually thereafter. At each visit we documented symptom evolution (daytime continence, urgency, enuresis, retention episodes), bowel status, voiding frequency, and adherence to timed voiding and pelvic floor exercises, together with urinalysis and urine culture when symptomatic. Ultrasound assessment included bladder wall appearance and post-void residual (PVR). Urodynamic reassessment was performed in cases of persistent symptoms, recurrent febrile UTIs, or significant post-void residuals on ultrasound, and after major therapeutic adjustments and is summarized longitudinally in Supplementary Table S1. Adherence to conservative measures (urotherapy and biofeedback home program) and to prescribed medication/CIC was recorded based on parental report and physiotherapy logs; recurrence in Case 1 after treatment withdrawal further supports the clinical relevance of sustained adherence.

Invasive studies (VCUG, cystoscopy) were not used as routine screening tests in our practice and were reserved for cases with anatomic suspicion; some procedures in these cases were performed before referral.

Clinical, imaging and urodynamic details of the three selected cases are summarized in Table 2.

This report does not represent a screened or consecutive cohort and was not designed to estimate incidence, prevalence, or outcome rates. Cases were selected for their illustrative value in highlighting severe, diagnostically challenging phenotypes. During the study period, most children referred for lower urinary tract dysfunction presented with mild to moderate forms responsive to standard urotherapy; only a small subset exhibited the severe, refractory phenotypes illustrated here.

This descriptive case series was prepared with reference to CARE reporting principles, adapted for a small illustrative case series, emphasizing transparent clinical description and longitudinal outcomes rather than population inference.

All data were handled in accordance with institutional ethical standards and the Declaration of Helsinki, and only anonymized information was used for analysis and publication.

A structured narrative review of the literature on severe LUTD and Hinman syndrome in neurologically normal children (1970–2024) was also performed using PubMed, Scopus, and Web of Science databases. Search terms combined MeSH and free-text keywords (“Hinman syndrome,” “non-neurogenic neurogenic bladder,” “functional lower urinary tract dysfunction,” “voiding dysfunction,” “children,” “pediatric”). Case reports, series, and observational studies meeting relevance criteria were included. Relevance criteria included: studies involving pediatric patients (≤18 years) with non-neurogenic functional LUTD or Hinman syndrome; articles providing clinical, urodynamic, imaging, or management data; publications in English; case reports, case series, or observational studies. Exclusion criteria were: reports focusing on neurogenic, anatomical, or postoperative bladder dysfunction; animal studies, conference abstracts without full data, or duplicates; studies lacking extractable clinical information. Reference lists of retrieved papers were considered to identify additional studies. Titles and abstracts were identified independently by two authors (O.S. and A.M.); disagreements were resolved by consensus. After applying these criteria, a small number of key publications—including the six most relevant clinical series and reviews summarized in Table 3—were selected to illustrate the historical and contemporary evidence base on severe functional LUTD in neurologically normal children.

### 2.2. Case Presentations

Case 1

A 7-year-old boy was referred for evaluation of recurrent febrile urinary tract infections (6 episodes in the previous year), persistent daytime incontinence, urgency and enuresis. He had normal perinatal history, developmental milestones, and no prior urinary tract abnormalities. Physical and neurological examinations were unremarkable, with no spinal or perineal anomalies. Ultrasound revealed moderate bladder wall thickening (Figure) and no post-void residual urine (PVR). Voiding cystourethrography (VCUG) illustrated a crenulated bladder contour, particularly during the voiding phase, and a mildly asymmetric (decentred) bladder configuration. No vesicoureteral reflux was identified. The posterior urethra appeared slightly dilated, yet bladder emptying was complete, with no post-void residual urine. Given the persistence of symptoms and recurrent infections, an exploratory cystoscopy was performed. The bladder was entered without difficulty. The bladder mucosa appeared markedly abnormal, displaying deep trabeculations with multiple cell-like and columnar ridges, giving a pseudo polypoid appearance. Small diverticula and dilated submucosal blood vessels were evident throughout the bladder wall. The extent of these mucosal changes rendered visualization of the ureteric orifices difficult. During withdrawal of the cystoscope, the bladder neck was noted to be high-set, while the verumontanum was normal, and the posterior urethra showed no signs of congenital posterior urethral valves. Urodynamic studies were consistent with detrusor overactivity with reduced bladder capacity (120 mL vs. expected 200 mL) and uninhibited contractions during the filling phase, supporting a diagnosis of severe functional lower urinary tract dysfunction.

The patient was started on standard urotherapy (timed voiding, adequate hydration, and bowel management) combined with trospium chloride (0.2 mg/kg twice daily) and continuous antibiotic prophylaxis with Nitrofurantoin (50 mg once daily). After four weeks, pelvic floor biofeedback training was introduced to improve sphincter coordination.

At six-month follow-up, he achieved full daytime continence with resolution of urgency and no new UTIs. Follow-up urodynamic assessment at 12 months showed improvement of bladder capacity and suppression of detrusor overactivity. Continuous antibiotic prophylaxis was withdrawn after 12 months, while trospium chloride was progressively tapered and discontinued at 24 months. Six months after discontinuation of all treatment, the patient developed a febrile urinary tract infection and recurrence of enuresis. The infection was treated with appropriate antibiotics, and combination therapy with trospium chloride and biofeedback was reinstated. At the last follow-up, 30 months after diagnosis, he remained continent and infection-free under maintenance urotherapy, with improved bladder capacity and stable urodynamic parameters.

In retrospect, the diagnostic sequence in this case might have been simplified. A DMSA renal scan and functional urodynamic assessment could have excluded significant reflux or obstruction without the need for VCUG. This case exemplifies how careful functional assessment can often replace invasive imaging when clinical and ultrasonographic findings do not suggest structural pathology.

Case 2

A 3-year-old girl presented to the Emergency Department (ED) of our tertiary pediatric referral center with fever and pollakiuria. Her history revealed that symptoms had begun two months earlier with dysuria and daytime incontinence, for which a urinary tract infection (UTI) had been diagnosed and treated with antibiotics. Despite appropriate therapy, her symptoms persisted. A renal and bladder ultrasound subsequently revealed left-sided ureterohydronephrosis, with dilatation of the renal pelvis and calyces and a crenulated bladder wall. In view of these findings and ongoing symptoms, the patient was referred to the Emergency Department for further evaluation and management. Voiding cystourethrography (VCUG): the study illustrated a grade V left-sided vesicoureteral reflux, with a dilated and tortuous ureter, dilated renal pelvis, and convex calyces. Voiding was not achieved during the examination. DMSA renal scintigraphy illustrated a small, irregularly contoured left kidney with marginal cortical scarring and thinned parenchyma, showing markedly reduced tracer uptake. The differential renal function was 81.3% for the right kidney and 18.7% for the left, indicating significant loss of function on the affected side. In this case, the patient underwent surgical correction with Cohen cross-trigonal ureteral reimplantation, performed through a standard open (transvesical) approach. Despite an initially favourable postoperative evolution, the patient developed recurrent febrile urinary tract infections and episodes of intermittent incontinence during medium- and long-term follow-up. Exploratory cystoscopy illustrated marked bladder mucosal remodelling, characterized by deep trabeculations and a coarse, cell-like and columnar architecture, rendering identification of the reimplanted ureteric orifices challenging. Following comprehensive evaluation and multidisciplinary case review, the patient was diagnosed with Hinman syndrome, representing a non-neurogenic neurogenic bladder secondary to severe functional voiding dysfunction.

Urodynamic evaluation was performed after resolution of urinary infection and bladder preparation. The filling cystometry illustrated reduced bladder compliance with early detrusor overactivity, producing sustained rises in intravesical pressure exceeding 40 cm H_2_O during the filling phase. Bladder sensation was present but inconsistent, and the patient reported urgency before reaching expected bladder capacity.

During the voiding phase, pressure–flow analysis revealed simultaneous detrusor contraction and external urethral sphincter activity, consistent with detrusor–sphincter dyssynergia. The uroflowmetry curve exhibited a staccato flow pattern, with low maximum flow rate (Qmax 5 mL/s) and prolonged voiding time. Post-void residual urine was elevated at 120 mL, indicating incomplete bladder emptying despite adequate detrusor effort. These urodynamic findings—poor compliance, detrusor overactivity, sphincter dyssynergia, and high post-void residuals—were highly suggestive of Hinman syndrome (non-neurogenic neurogenic bladder), correlating with the patient’s clinical picture of recurrent febrile urinary tract infections and incontinence. The patient was started on a comprehensive treatment regimen that included continuous antibiotic prophylaxis (CAP), trospium chloride, clean intermittent catheterization (CIC), and pelvic floor biofeedback therapy. Clinical evolution was slowly favourable, with progressive improvement in bladder function and complete resolution of recurrent urinary tract infections over time. At 36-month follow-up, she maintained normal voiding intervals and remained free of febrile UTIs, with stable renal function on DMSA and no need for further surgical intervention.

This case was retained in our series to exemplify a diagnostic pitfall—namely, severe functional voiding disorder masquerading as primary structural reflux, resulting in persistent symptoms after anatomically successful ureteral reimplantation. The prior decision to perform ureteral reimplantation was made before referral and likely preceded full recognition of the underlying functional bladder dysfunction. In hindsight, this case illustrates the diagnostic pitfall of attributing high-grade reflux solely to structural causes. Vesicostomy was considered as a theoretical option for temporary decompression but was ultimately unnecessary, as combined CIC, antimuscarinic therapy, and biofeedback achieved satisfactory bladder emptying and infection control. Uroflowmetry, though performed in a three-year-old with incomplete toilet training, was interpreted cautiously and complemented by invasive urodynamic testing to confirm detrusor–sphincter dyssynergia.

Case 3

A 10-year-old boy presented to the Emergency Department (ED) of our tertiary pediatric referral center with acute urinary retention. He had a total of three prior emergency presentations for similar symptoms before being admitted for comprehensive evaluation and further investigations. Renal and bladder ultrasound: The urinary bladder, evaluated in a partially filled state, showed marked wall thickening (up to 7–8 mm) with an irregular inner contour and heterogeneous intravesical content, suggestive of chronic inflammatory changes. Voiding cystourethrography (VCUG): The urinary bladder illustrated a large capacity with a slightly crenulated contour and uniform opacification. A grade I vesicoureteral reflux was noted. Voiding was difficult, intermittent, and incomplete, with a significant post-void residual volume. The urethra appeared normal throughout its course.

Urodynamic studies: The evaluation illustrated an underactive detrusor, with a maximum urinary flow rate of 4 mL/s and a weak, low-pressure urinary stream. A significant post-void residual volume was recorded, consistent with incomplete bladder emptying.

Exploratory cystoscopy: The bladder was entered without difficulty. The bladder mucosa appeared markedly altered, showing cellular and columnar ridges with pronounced trabeculation and a pseudo-diverticular appearance at the trigone. The posterior urethra was normal, with no evidence of anatomical obstruction. The patient was treated with tamsulosin (Omnic-Tocas) and pelvic floor biofeedback therapy, resulting in a gradually favourable clinical evolution and complete resolution of acute urinary retention episodes. In this case, the initial suspicion of posterior urethral valves justified both VCUG and cystoscopy, underscoring the selective use of invasive imaging when structural obstruction is part of the differential diagnosis.

At 24-month follow-up, the patient continued to void spontaneously without residual urine or retention episodes, and follow-up ultrasound showed normalized bladder wall thickness.

Summary of Patient Characteristics:

The three pediatric patients (two males, one female; aged 3–10 years) presented with severe lower urinary tract dysfunction (LUTD) manifesting as recurrent febrile urinary tract infections, incontinence, or urinary retention, in the absence of neurological or structural abnormalities.

Across the three cases, recurrent and often persistent urinary tract infections (UTIs) were a major clinical feature, closely reflecting the severity of the underlying lower urinary tract dysfunction. *Escherichia coli* was isolated in two patients and *Klebsiella pneumoniae* in one, consistent with the typical uropathogen spectrum in pediatric LUTD. Several episodes involved recurrent bacteriuria with the same strain, suggesting incomplete bladder emptying and biofilm persistence rather than reinfection. One *E. coli* isolate exhibited extended-spectrum β-lactamase (ESBL) production, and two isolates showed resistance to ampicillin and first-generation cephalosporins, highlighting the risk of antimicrobial resistance in children with chronic functional stasis. All infections responded to targeted antibiotic therapy guided by culture and sensitivity testing, combined with optimization of bladder function through urotherapy and pharmacologic modulation. Importantly, no further febrile or culture-proven UTIs occurred once effective bladder emptying was achieved and infection control measures were maintained, underscoring the link between recurrent infection and underlying functional obstruction.

**Figure 1:**
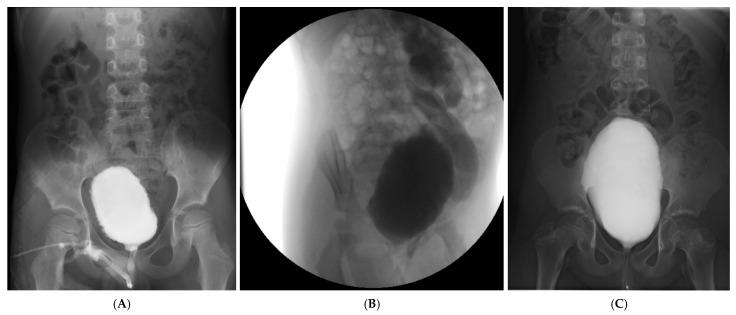
VCUG images. Representative imaging and endoscopic findings from the three pediatric patients with severe functional LUTD and recurrent urinary tract infections (**A**–**C**). (**A**) Case 1: Voiding cystourethrography showing a crenulated, asymmetric bladder contour without reflux; (**B**) Case 2: Voiding cystourethrography illustrating grade V left vesicoureteral reflux with a tortuous, dilated ureter and dilated pelvis and calyces; (**C**) Case 3: Voiding cystourethrography depicting a large-capacity bladder with mild crenulation and grade I reflux, associated with incomplete voiding and high post-void residual.

Imaging and urodynamic evaluations revealed diverse functional patterns ranging from detrusor overactivity and detrusor–sphincter dyssynergia to underactivity, each associated with bladder wall remodeling evident on cystoscopy (trabeculation, pseudopolypoid mucosa, diverticula) (Figure 1).

All patients underwent multimodal management, including combinations of urotherapy, anticholinergic or α-blocker therapy, biofeedback, and in selected cases continuous antibiotic prophylaxis or clean intermittent catheterization. Clinical outcomes were favorable in all cases, with resolution of infections and improvement in continence or bladder emptying, although one patient (Case 1) experienced recurrence after discontinuation of therapy.

To facilitate comparison across patients, the key urodynamic variables and imaging findings for each case are summarized in Table 2, including bladder capacity, compliance, detrusor activity, presence of detrusor–sphincter dyssynergia, and post-void residual volume. In addition, Supplementary Table S1 provides a longitudinal overview of urodynamic evolution during follow-up, illustrating changes in bladder dynamics and residual urine volumes in response to conservative management. Medium-term clinical outcomes, post-void residual volumes, urodynamic evolution, and treatment adherence are summarized in Supplementary Table S2. Quantitative changes in bladder capacity, detrusor pressure, and post-void residual volumes before and after treatment are summarized for all cases in Supplementary Tables S1 and S2.

**Table 2:** Summary Table of Cases.

Characteristic	Case 1	Case 2	Case 3
Age/Sex	7 years/Male	3 years/Female	10 years/Male
Presenting Symptoms	Recurrent febrile UTIs, daytime incontinence, urgency, enuresis	Fever, pollakiuria, dysuria, incontinence	Acute urinary retention (recurrent episodes)
Prior History	Multiple UTIs over the preceding year	Persistent LUTS after treated UTI	Three prior ED visits for retention
Ultrasound Findings	Moderate bladder wall thickening; no residual urine	Left ureterohydronephrosis; crenulated bladder wall	Bladder wall thickened (7–8 mm), irregular contour, heterogeneous content
VCUG Findings	Crenulated bladder, no reflux, normal emptying	Grade V left VUR, dilated tortuous ureter, no voiding achieved	Large-capacity bladder, mild crenulation, grade I VUR, incomplete emptying
Renal Scintigraphy (DMSA)	–	Left kidney: small, irregular, cortical scarring; function 18.7%	–
Cystoscopy Findings	Deep trabeculations, pseudopolypoid mucosa, diverticula, dilated vessels	Marked trabeculation, cell-like and columnar mucosa, difficult ureteric visualization	Trabeculated, pseudodiverticular bladder mucosa, normal urethra
Urodynamic Pattern	Detrusor overactivity, reduced capacity, uninhibited contractions	Poor compliance, detrusor–sphincter dyssynergia, high PVR	Hypocontractile detrusor, weak flow (Qmax 4 mL/s), high PVR
Diagnosis	Severe functional LUTD	Hinman syndrome (non-neurogenic neurogenic bladder)	Functional LUTD with hypocontractile bladder
Treatment	Urotherapy, trospium chloride, CAP, biofeedback	CAP, trospium chloride, CIC, biofeedback	Tamsulosin (Omnic-Tocas), biofeedback
Outcome	Initial remission; recurrence after withdrawal, improved with retreatment	Gradual improvement; resolution of UTIs, stable bladder function	Gradual recovery; resolution of acute retention episodes

## 3. Discussion

Severe lower urinary tract dysfunction (LUTD) in neurologically and anatomically normal children remains a diagnostic and therapeutic challenge. While mild functional disorders such as overactive bladder or dysfunctional voiding are common in paediatrics, severe presentations involving high-pressure voiding, recurrent febrile urinary tract infections (UTIs), and bladder wall remodelling are rare. The three cases presented here illustrate distinct phenotypes within the spectrum of severe functional LUTD, ranging from detrusor overactivity and detrusor–sphincter dyssynergia to detrusor underactivity, all occurring in neurologically intact children without structural obstruction.

The diagnostic procedures described in these cases (such as voiding cystourethrography and cystoscopy) were conducted before referral to our department and reflect prior diagnostic workups rather than our recommended initial approach. In current practice, evaluation of lower urinary tract dysfunction in neurologically and anatomically normal children should prioritize noninvasive and functional assessments, including uroflowmetry, ultrasound-based post-void residual measurement, and, when appropriate, renal scintigraphy. Invasive studies such as VCUG or cystoscopy should be reserved for cases with clinical or imaging features suggesting an anatomic abnormality. This distinction reinforces the importance of a “function-first” diagnostic strategy. The apparent discrepancy between our critique of unnecessary invasive testing and the use of VCUG and cystoscopy in all three cases reflects the retrospective nature of this series and referral-based practice rather than a contradiction in diagnostic philosophy. In two cases, invasive imaging was performed before referral, in response to suspected structural pathology (recurrent febrile UTIs, hydronephrosis, or concern for posterior urethral valves). In the third case, VCUG and cystoscopy were justified by acute urinary retention and the need to exclude anatomical obstruction. Importantly, these investigations were exclusionary rather than confirmatory, serving to rule out structural causes when clinical presentation or non-invasive imaging raised concern.

### 3.1. Suggested Diagnostic Approach to Severe LUTD in Neurologically Intact Children

Based on our clinical experience and the cases presented, evaluation of severe LUTD in neurologically intact children should follow a stepwise, function-first approach. Initial assessment should include detailed history, bladder and bowel diary, physical and neurological examination, renal and bladder ultrasound, and ultrasound-based post-void residual (PVR) measurement. Non-invasive uroflowmetry (with or without surface EMG when available) should be performed to identify voiding patterns suggestive of dysfunction.

Invasive investigations such as urodynamic testing are reserved for children with severe or refractory symptoms, recurrent febrile UTIs, elevated PVR, or suspected high-pressure voiding. VCUG should be considered only when reflux or anatomical obstruction is suspected based on ultrasound or clinical presentation. Cystoscopy should be reserved for cases with inconclusive imaging, suspicion of urethral obstruction, or persistent symptoms despite appropriate functional therapy. This algorithm prioritizes functional assessment while limiting invasive procedures to situations where exclusion of structural pathology is clinically justified.

### 3.2. Pathophysiologic Considerations

The pathophysiology of severe functional LUTD in such patients remains incompletely understood. Historically, similar presentations have been described under the term Hinman syndrome, or non-neurogenic neurogenic bladder, first characterized by Hinman and Bauman as a functional voiding disorder in which children develop detrusor–sphincter discoordination and bladder wall changes typical of neurogenic bladder, but without any detectable neurological lesion [12,13]. In this condition, children exhibit the urodynamic and morphological characteristics of neurogenic bladder—such as detrusor–sphincter dyssynergia, trabeculated bladder wall, and progressive upper urinary tract changes—yet lack any identifiable neurological lesion [13,14,15]. The underlying mechanism is believed to involve maladaptive learned voiding behaviors, chronic contraction of the external urethral sphincter during voiding, and consequent elevation of intravesical pressures. Over time, these functional disturbances lead to detrusor hypertrophy, loss of compliance, and secondary vesicoureteral reflux, closely mirroring the cystoscopy and urodynamic findings observed in our cohort [2,7].

Recent evidence suggests that Hinman syndrome may represent the severe end of the non-neurogenic LUTD spectrum, wherein prolonged behavioral dysfunction, emotional stressors, and delayed diagnosis culminate in irreversible bladder remodeling and renal compromise [16,17]. Recognition of this continuum is essential, as early identification and targeted functional therapy—combining urotherapy, biofeedback, pharmacologic modulation (e.g., trospium chloride), and psychological support—can halt or even reverse detrusor deterioration in many cases [18]. Conversely, misinterpretation of these findings as structural pathology and subsequent surgical interventions, such as reimplantation or bladder outlet procedures, frequently result in persistence or worsening of symptoms. Therefore, awareness of the Hinman paradigm reinforces the need for a function-first diagnostic approach and comprehensive multidisciplinary management to prevent progression to upper tract damage and preserve long-term renal function.

Chronic maladaptive voiding behaviour—often secondary to voluntary withholding, pain, or psychosocial stressors—can lead to persistently elevated detrusor pressures, incomplete emptying, and bladder remodelling. Over time, the resulting high-pressure bladder dynamics can induce trabeculation, pseudodiverticula, and mucosal vascular congestion, as observed cystoscopically in all three of our patients. Hinman syndrome can be regarded as the severe endpoint of the functional lower urinary tract dysfunction (LUTD) continuum. It represents the stage at which chronic maladaptive voiding patterns, sustained detrusor–sphincter dyssynergia, and persistent outlet obstruction culminate in structural bladder remodeling, loss of compliance, and upper tract risk, despite the absence of neurological or anatomical disease. In this framework, less severe entities—such as overactive bladder, dysfunctional voiding, and underactive bladder—reflect earlier or compensatory phases within the same functional spectrum. Recognizing this progression emphasizes the need for early diagnosis and targeted urotherapy to prevent irreversible detrusor and renal damage.

### 3.3. Clinical and Diagnostic Correlation

In our series, Case 1 presented with severe detrusor overactivity and reduced bladder capacity, leading to recurrent UTIs and incontinence, but with preserved bladder emptying. Case 2 illustrated the classical features of Hinman syndrome, including detrusor–sphincter dyssynergia, poor compliance, and secondary high-grade vesicoureteral reflux (VUR), ultimately resulting in unilateral renal scarring and loss of function. Case 3, conversely, displayed a hypo contractile detrusor and high residual volumes, suggesting a late-stage, decompensated functional lower urinary tract dysfunction (LUTD). Despite these distinct urodynamic profiles, all cases shared endoscopic evidence of severe bladder wall remodeling—deep trabeculations, pseudo polypoid mucosa, and submucosal vascular changes—supporting a continuum of functional injury resulting from chronic dyscoordination of voiding. Cystoscopy features such as trabeculation, pseudopolypoid mucosa, diverticula, and submucosal vascular congestion are not disease-specific and may be observed in neurogenic bladder, chronic bladder outlet obstruction, or long-standing functional voiding disorders. In the present series, these findings were interpreted in conjunction with urodynamic patterns, imaging, neurological examination, and clinical evolution, rather than as standalone diagnostic markers. Their relevance lies in reflecting chronic exposure to elevated intravesical pressures and functional obstruction rather than defining a specific etiology. In all three cases, differential diagnoses included posterior urethral valves, urethral stricture, primary bladder neck obstruction, and occult neurogenic bladder. These were excluded based on combined findings from VCUG, cystoscopy, urodynamics, neurological examination, and clinical follow-up. The absence of anatomical obstruction, normal urethral anatomy, and sustained improvement under functional therapy supported a functional etiology. Although histologic correlation was not available, clinical response to conservative management further argued against fixed structural obstruction.

Exploratory cystoscopy played a corroborative and exclusionary role, primarily allowing exclusion of anatomical obstruction rather than providing disease-specific diagnostic features. The absence of these findings, combined with characteristic urodynamic patterns, was consistent with functional etiology in all patients. This reinforces the importance of a structured diagnostic algorithm combining imaging, urodynamics, and endoscopic assessment in children with severe, atypical LUTD.

Hinman syndrome is defined by (1) detrusor–sphincter dyssynergia with high voiding pressures, (2) poor bladder compliance, (3) characteristic bladder wall trabeculation or pseudodiverticula, (4) recurrent UTIs/VUR, and (5) absence of neurological or anatomical lesions [13]. Case 2 fulfilled all five criteria, confirming classical Hinman syndrome. In contrast, Cases 1 and 3 displayed partial phenotypes—overactivity with preserved compliance and underactivity respectively—representing milder or compensatory forms within the same functional spectrum.

The findings of this case series are consistent with published reports on severe non-neurogenic LUTD and Hinman syndrome, particularly regarding urodynamic abnormalities, bladder wall remodeling, and the risk of recurrent infection and upper tract involvement. Case 2 closely reflects classical descriptions of Hinman syndrome, with poor compliance, detrusor–sphincter dyssynergia, secondary high-grade reflux, and highlighted renal damage, highlighting a well-recognized diagnostic pitfall in which functional obstruction is misinterpreted as primary structural pathology. In contrast, Cases 1 and 3 illustrate distinct but less frequently emphasized phenotypes within the same functional continuum—severe detrusor overactivity with preserved emptying and detrusor underactivity with retention—both capable of producing significant bladder remodeling and infectious morbidity. Unlike many historical series reporting progressive renal deterioration, all patients in the present cohort achieved stabilization or improvement with conservative, function-oriented management, likely reflecting earlier recognition, systematic urodynamic phenotyping, and contemporary multimodal therapy. The relapse observed after treatment withdrawal in Case 1 further supports the dynamic, behavior-dependent nature of these disorders. Collectively, these observations support viewing severe functional LUTD and Hinman syndrome as a spectrum rather than discrete entities and reinforce current recommendations to prioritize functional assessment and conservative management to modify disease trajectory and avoid unnecessary surgery.

While occult neurogenic causes cannot be entirely excluded without MRI or EMG, the consistent clinical course and response to functional therapy across all cases support a predominantly functional etiology.

### 3.4. Management Implications

Management of severe functional LUTD requires a multimodal approach, addressing both bladder dynamics and behavioural contributors. In all three patients, therapy was based on the integration of pharmacologic modulation, pelvic floor retraining (biofeedback), and, when indicated, continuous antibiotic prophylaxis (CAP) or clean intermittent catheterization (CIC). In our institution, trospium use in children is considered off-label but follows institutional pediatric urology protocols and parental informed consent in line with national regulatory guidance.

Tamsulosin, used in Case 3, has been reported in small pediatric cohorts to reduce functional outlet resistance and improve emptying in selected cases of detrusor underactivity and voiding dysfunction [2,19].

Anticholinergic therapy with trospium chloride, used in Cases 1 and 2, effectively reduced detrusor overactivity and improved bladder compliance, consistent with previously reported paediatric outcomes. In Case 3, tamsulosin (Omnic-Tocas) was chosen to reduce functional outlet resistance and facilitate voiding in the setting of a hypo contractile bladder. Biofeedback training proved beneficial across all cases, enhancing sphincter relaxation and voiding coordination, which are often impaired in these children. The gradual but consistent clinical improvement, including resolution of recurrent UTIs and continence restoration, highlights the efficacy of this non-surgical, function-oriented therapy.

The therapeutic use of trospium chloride in children with lower urinary tract dysfunction has been increasingly documented, although data remain limited compared to adult populations. Controlled studies, such as that by Pereira et al. on children with detrusor overactivity, illustrated significant symptomatic and urodynamic improvement with trospium compared to placebo, supporting its efficacy in paediatric overactive bladder [20]. Subsequent reviews and clinical overviews have confirmed its favourable safety profile and rapid therapeutic response, with up to 90% of children responding within the first week of treatment [21]. Importantly, trospium’s quaternary ammonium structure limits its penetration across the blood–brain barrier, resulting in fewer central nervous system adverse effects relative to other antimuscarinics, a key advantage in paediatric use [22,23].

Although most published studies involve idiopathic or neurogenic detrusor overactivity, rather than severe functional LUTD or Hinman syndrome, their findings support the pharmacologic rationale for antimuscarinic therapy in such cases. In our series, trospium chloride was combined with biofeedback-based pelvic floor retraining, leading to marked symptomatic improvement and resolution of infections in all three patients. These outcomes align with prior evidence suggesting that bladder relaxation, improved compliance, and reduction in detrusor overactivity are achievable in children when trospium is used as part of a structured conservative regimen [20,21,22,23]. Given the paucity of reports in severe, non-neurogenic LUTD, our experience expands on the existing literature by illustrating that trospium chloride may play a useful adjunctive role in preventing recurrent urinary tract infections and preserving bladder compliance in this complex subgroup.

### 3.5. Combination Therapy with Trospium Chloride and Biofeedback

In our institution, we have increasingly adopted a combination of biofeedback training and pharmacologic modulation with trospium chloride for children presenting with severe non-neurogenic LUTD, particularly those with detrusor overactivity or dysfunctional voiding unresponsive to standard urotherapy alone. Trospium chloride, a quaternary ammonium antimuscarinic, exhibits limited central nervous system penetration due to its hydrophilic nature, making it particularly well-suited for pediatric patients who may experience cognitive or behavioral side effects with tertiary amines such as oxybutynin. In our three cases, trospium chloride was introduced (at a dose of 0.2–0.3 mg/kg twice daily) in two patients showing persistent urgency and detrusor overactivity despite initial urotherapy. Both patients underwent concurrent pelvic floor biofeedback sessions, emphasizing relaxation training, sphincter coordination, and timed voiding strategies. Within 6 to 8 weeks, both children illustrated marked symptomatic improvement—reduction in daytime incontinence episodes, improved bladder capacity on follow-up uroflowmetry, and complete resolution of recurrent urinary tract infections. No significant anticholinergic side effects (dry mouth, constipation, or blurred vision) were observed during treatment. The synergistic use of biofeedback and trospium chloride appears to enhance therapeutic response by combining pharmacologic reduction in detrusor overactivity with behavioral retraining of pelvic floor control. Similar results have been reported in recent pediatric cohorts, where biofeedback-based urotherapy augmented by selective antimuscarinics significantly improved bladder compliance and reduced infection recurrence rates. These outcomes reinforce the value of a multimodal, function-focused approach in children with refractory functional LUTD before considering invasive procedures.

Trospium chloride offers a distinct pharmacological advantage in pediatric LUTD management due to its limited central nervous system penetration, favorable safety profile, and complementary effect with behavioral retraining via biofeedback. This combination allows functional restoration without the neurocognitive side effects sometimes seen with tertiary amines, making it a preferred option in our institutional protocol for severe or refractory functional LUTD.

### 3.6. Outcomes and Comparison with Literature

The structured narrative review identified key publications that contextualize our findings within the broader evidence on severe functional LUTD in neurologically normal children (Table 3). To complement the qualitative descriptions presented in the Results, Supplementary Table S1 summarizes the baseline and follow-up urodynamic characteristics for each case. Regular monitoring of renal function (serum creatinine and ultrasound) was consistent with preserved upper tract integrity and underscores the importance of incorporating kidney function evaluation into the management of children with severe LUTD.

All three patients fit well within the functional–non-neurogenic spectrum described in prior reports of Hinman syndrome and severe LUTD. Like historical cases, all illustrated bladder wall trabeculation and abnormal voiding dynamics without neurogenic lesions. However, unlike many earlier series where renal impairment was common, timely recognition and functional management (biofeedback, pharmacologic modulation, CAP/CIC) in your cases led to favorable outcomes and renal preservation. This underscores the evolution in diagnostic awareness and conservative treatment efficacy over the past two decades (Table 3).

With early diagnosis and appropriate multidisciplinary management, functional recovery and renal preservation are achievable. All three patients in our series illustrated significant clinical improvement and stabilization of the upper urinary tract following conservative therapy. However, recurrence after withdrawal of treatment, as seen in Case 1, highlights the need for long-term follow-up and patient adherence to urotherapy and behavioral measures. In contrast, delayed recognition and inappropriate surgical management, as illustrated by Case 2, may contribute to irreversible renal damage.

The clinical spectrum and outcomes of severe functional lower urinary tract dysfunction (LUTD) and Hinman syndrome have been documented in several case reports and small series over the past five decades. The original description by Hinman and Bauman highlighted the concept of a non-neurogenic neurogenic bladder, in which chronic functional obstruction and detrusor–sphincter discoordination produce bladder wall trabeculation, diverticula, and progressive renal injury despite normal neurological findings [13]. Subsequent studies, including Lee et al. (2007), reported long-term follow-up of 14 pediatric cases, illustrating that poor bladder compliance and persistent high detrusor pressures are common and may lead to vesicoureteral reflux (VUR) and upper tract deterioration if untreated [24]. Jayanthi et al. (1997) expanded the understanding of this entity to infancy, describing similar urodynamic findings even before toilet training [25].

**Table 3:** Comparison of Present Cases with Published Reports on Severe Functional Lower Urinary Tract Dysfunction and Hinman Syndrome.

Study/Source	Age/Sex	Neurological Findings	Key Imaging/Cystoscopic Findings	Urodynamic Pattern	Management	Outcome/Remarks
Present Report—Case 1	7 y/M	Normal	Crenulated bladder, trabeculated mucosa, pseudopolypoid changes	Detrusor overactivity, small capacity, uninhibited contractions	Trospium chloride, CAP, biofeedback	Resolution of incontinence and UTIs; relapse after withdrawal, improved with retreatment
Present Report—Case 2	3 y/F	Normal	Grade V VUR, trabeculated pseudopolypoid mucosa, difficult ureteric visualization	Poor compliance, detrusor–sphincter dyssynergia, high PVR	CAP, trospium chloride, CIC, biofeedback	Gradual functional improvement; resolution of UTIs; diagnosed as Hinman syndrome
Present Report—Case 3	10 y/M	Normal	Large-capacity bladder, mild crenulation, trabeculated trigone	Hypocontractile detrusor, low Qmax (4 mL/s), high PVR	Tamsulosin (Omnic-Tocas), biofeedback	Slow but complete resolution of acute retention
Hinman [13]	6–15 y/Mixed	Normal	Trabeculated, thick-walled bladder; VUR common	Detrusor–sphincter dyssynergia; high pressures	Behavioral retraining, catheterization	Variable; some progressed to renal failure
Lee et al. [24]	5–17 y/14 pts	Normal	Trabeculation, diverticula, VUR in 50%	Poor compliance; DSD	CIC ± anticholinergics	Improved bladder compliance; preserved renal function in most
Chaichanamongkol et al. [26]	1.5 y/M	Normal	VUR, hydronephrosis	DSD; poor compliance	CIC, CAP	Recovery from renal failure; long-term follow-up stable
Gampala et al. [12]	14 y/M	Normal	Bilateral VUR, trabeculated bladder	DSD; incomplete voiding	Anticholinergic, CIC	Improved voiding and infection control
Jayanthi et al. [25]	<2 y/Mixed	Normal	Thickened bladder wall; reflux	DSD, poor compliance	CIC, behavioral therapy	Early infancy presentation; good outcome with early management
Wan et al. [27]	9 y/F	Normal	Normal bladder and urethra	Normal detrusor; voluntary retention	Psychological counseling	Complete recovery; illustrates differential

Individual case reports, such as those by Chaichanamongkol et al. (2008) and Gampala et al. (2024), reinforced that Hinman syndrome can manifest with recurrent urinary retention, hydronephrosis, and even renal failure, but that timely initiation of clean intermittent catheterization (CIC) and pharmacologic therapy can stabilize or reverse renal dysfunction [12,26]. Others emphasized the role of detailed urodynamic assessment in distinguishing functional from neurogenic causes of LUTD and advocated early behavioral and biofeedback-based interventions to improve detrusor–sphincter coordination [16]. In addition, rare presentations of psychogenic urinary retention in otherwise normal children, such as that reported by Wan and Yang (2010), highlight the potential contribution of psychosocial and learned behavioral factors in this functional spectrum [27].

Overall, the literature supports the interpretation that severe LUTD in neurologically intact children represents a continuum ranging from dysfunctional voiding to the full expression of Hinman syndrome, with outcomes closely linked to the timing and adequacy of diagnosis. Early multidisciplinary intervention—combining urotherapy, pharmacologic modulation, and biofeedback retraining—remains the cornerstone of successful management and prevention of irreversible renal damage.

In comparison with previously published reports, our three cases share several key features with the classical descriptions of severe non-neurogenic LUTD and Hinman syndrome. Similarly to the cohorts reported by Hinman and Lee, all our patients exhibited detrusor–sphincter discoordination, bladder wall trabeculation, and recurrent febrile urinary tract infections despite the absence of neurological lesions [13,24]. However, unlike many historical series in which delayed diagnosis led to progressive renal impairment, none of the children in our cohort developed irreversible upper tract damage. This difference likely reflects the benefits of early functional assessment, urodynamic phenotyping, and structured conservative management, including biofeedback and pharmacologic modulation, which are now increasingly emphasized in contemporary pediatric urology guidelines.

The present case series contributes to existing evidence by illustrating the functional diversity and reversibility of severe LUTD in neurologically intact children. Whereas prior studies often described Hinman syndrome as a fixed, late-stage entity, our findings support the concept of a spectrum of severity within non-neurogenic functional LUTD—encompassing overactive, dyssynergy, and hypo-contractile phenotypes—all capable of meaningful recovery under a multimodal, function-first therapeutic strategy. Furthermore, the close correlation observed between remodeling and urodynamic patterns reinforces the idea that bladder wall changes can occur secondary to functional obstruction rather than primary structural disease. Recognizing this relationship helps clinicians avoid unnecessary surgical interventions and underscores the value of early conservative management in preserving renal function and long-term continence outcomes.

### 3.7. Misdiagnosis and Iatrogenic Management Pitfalls

An important observation from both our experience and the published literature is that children with severe lower urinary tract dysfunction (LUTD) are often misdiagnosed as having structural abnormalities, leading to unnecessary surgical interventions such as endoscopic bulking injections or ureteral reimplantation [28,29]. These procedures are frequently undertaken in response to recurrent urinary tract infections (UTIs) or hydronephrosis, without comprehensive evaluation of underlying voiding dysfunction. When the aetiology is functional LUTD outlet obstruction or detrusor overactivity, such surgeries fail to address the primary pathophysiology. Consequently, incontinence, recurrent infection, and high post-void residuals may persist postoperatively, and upper tract deterioration can progress due to sustained high intravesical pressures [28,29,30].

Several studies show that persistent VUR or postoperative febrile UTIs after anti-reflux surgery often reflect unrecognized bladder–bowel dysfunction (BBD)/dysfunctional voiding (DV) rather than primary surgical failure. Classic reimplantation series linked reimplantation failure and postoperative problems to DV [31]. Whittam et al. found that postoperative febrile UTI after ureteroneocystostomy was significantly associated with dysfunctional elimination syndrome [32]. After bilateral extravesical reimplantation, postoperative voiding dysfunction is well documented [33,34], and the broader literature and guidelines emphasize that untreated BBD worsens outcomes and should be managed before anti-reflux procedures [35,36].

The International Children’s Continence Society (ICCS) guidelines further caution against performing anti-reflux or outlet procedures before comprehensive functional assessment, including uroflowmetry, post-void residual measurement, and urodynamic studies [2,37]. Misinterpreting incontinence or recurrent infections as indicators of anatomical reflux rather than functional dysfunction remains a leading cause of iatrogenic persistence of symptoms and poor long-term outcomes [2].

In our cohort, all three patients were initially assessed elsewhere for suspected reflux or obstruction, and two had been considered for cystoscopy intervention before referral. Correct recognition of non-neurogenic functional LUTD and implementation of urotherapy-based management led to marked symptom improvement or complete resolution, thereby avoiding unnecessary surgical procedures. Case 2 particularly illustrates this pitfall: the patient underwent Cohen cross-trigonal ureteral reimplantation for presumed reflux, yet continued to experience infections and incontinence until a diagnosis of Hinman syndrome was made. Similar outcomes have been described in other reports, where endoscopic bulking, bladder neck incisions, or reimplantation failed to yield durable benefit due to underlying functional voiding disorders [28,29,30]. Collectively, these findings underscore that in children with severe LUTD, surgical correction should be deferred until functional aetiologies are rigorously excluded, and a function-first, multidisciplinary diagnostic paradigm should be adopted to prevent iatrogenic morbidity [1,37,38].

Case 1 illustrates that in retrospect, non-invasive functional assessment could have preceded VCUG, whereas in Case 3, acute urinary retention justified invasive imaging to exclude obstruction, underscoring the importance of individualized, indication-driven testing.

Common Missteps in the management of LUTD in Otherwise healthy children are summarized in Table 4 along with alternative approach considering current guidelines. 

### 3.8. Limitations

As a purposively selected illustrative case series, this report is inherently subject to selection bias and is not intended to support population-level inference.

Our series is small and retrospective, limiting generalizability. We did not employ validated symptom instruments (e.g., DVSS/Vancouver) at every visit, nor did we include a control group; future prospective studies should incorporate standardized patient-reported outcomes, rigorous UTI definitions, and longer renal follow-up. Imaging and urodynamics were performed at a tertiary center, which may introduce referral bias.

Another limitation is the absence of systematic neurophysiological testing or spinal imaging (e.g., EMG or spinal MRI). Although all patients underwent repeated, comprehensive neurological examinations with normal findings and showed no clinical features suggestive of occult neurogenic pathology, subclinical spinal abnormalities cannot be fully excluded. However, current pediatric urology practice generally reserves advanced neuroimaging for children with suggestive symptoms or examination abnormalities, and none were present in this series.

Quantitative urodynamic data were not consistently available for all follow-up visits; therefore, a summarized overview is provided as Supplementary Table S1 to illustrate the trends in bladder compliance, detrusor pressure, and post-void residual improvement. Raw urodynamic tracings were not uniformly available for publication; however, standardized reports and quantitative parameters were reviewed and are summarized in Table 2 and Tables S1 and S2.

Histologic sampling of bladder mucosa was not performed, as cystoscopy was used solely for diagnostic exclusion rather than research purposes; therefore, interpretation of mucosal changes relied on functional and clinical correlation rather than histopathology.

As with all retrospective pediatric studies, ethical safeguards relied on prior institutional consent and strict data anonymization rather than prospective assent procedures.

### 3.9. Clinical Takeaways

Severe LUTD in otherwise healthy children is not rare in specialty practice and should be considered when rUTIs persist despite guideline-based infection management. (2) Early urodynamic phenotyping is valuable to direct therapy. (3) Urotherapy plus targeted add-ons (biofeedback, pharmacotherapy, CIC) can meaningfully reduce infections and protect the upper tracts. (4) Consistent use of ICCS terminology facilitates care coordination and research comparability.

The three cases presented reinforce the growing body of evidence that severe lower urinary tract dysfunction (LUTD) can occur in neurologically and anatomically normal children, representing the functional spectrum of disorders historically encompassed by Hinman syndrome. Despite differences in urodynamic profiles—ranging from detrusor overactivity to underactivity—all patients exhibited high-pressure bladder dynamics and secondary bladder wall remodeling, which are recognized risk factors for upper urinary tract deterioration and renal scarring if left untreated. Early recognition of this condition, supported by comprehensive urodynamic assessment and endoscopic evaluation, is therefore essential to distinguish functional from anatomical causes of obstruction. Moreover, our experience underscores that timely initiation of conservative, function-oriented management—including pharmacologic modulation, biofeedback retraining, and, when necessary, intermittent catheterization—can lead to substantial functional recovery and preservation of renal integrity. These observations highlight the importance of multidisciplinary follow-up and long-term behavioral reinforcement in achieving sustained remission and preventing irreversible renal damage.

Collectively, these observations extend current knowledge by illustrating that even the most severe forms of functional LUTD can achieve sustained remission when identified early and treated through coordinated, multidisciplinary care.

## 4. Conclusions

These cases collectively illustrate the heterogeneity and clinical complexity of severe functional LUTD in neurologically normal children. Recognition of this disorder as part of the Hinman spectrum is essential to avoid unnecessary surgical interventions and to implement early, targeted urodynamic and behavioral therapy. Multidisciplinary evaluation—including pediatric urology, nephrology, physiotherapy, and psychology—is critical to achieve durable functional recovery and prevent long-term renal morbidity. The recommendation to prioritize functional assessment and avoid premature surgical intervention is directly supported by the favorable clinical and urodynamic outcomes observed in all three patients.

## Figures and Tables

**Table 1:** Shared Clinical Characteristics of Reported Cases.

Characteristic	Description/Rationale
Neurologically and anatomically normal	Supports non-neurogenic, non-structural etiology
Recurrent UTIs (≥2 febrile or ≥3 culture-proven within 12 months)	Indicates clinically significant morbidity
Urodynamic evidence of detrusor overactivity, dyssynergia, or underactivity	Defines severe functional LUTD phenotype
No prior lower urinary tract surgery related to functional obstruction at the time of LUTD evaluation; no systemic disease affecting voiding	Excludes iatrogenic or systemic confounders

**Table 4:** Common Missteps in the Management of Severe Lower Urinary Tract Dysfunction (LUTD) in Otherwise Healthy Children.

Common Misstep	Underlying Issue/Reason	Typical Consequence	Evidence-Based Alternative (ICCS & Current Guidelines) *
Performing cystoscopic bulking injections for presumed VUR without functional assessment	Misinterpretation of rUTIs or hydronephrosis as anatomical reflux	Persistence or recurrence of UTIs and incontinence; unresolved high bladder pressures; possible upper tract deterioration	Comprehensive LUTD work-up first: uroflowmetry, post-void residual (PVR), and urodynamics; initiate urotherapy ± pharmacotherapy before considering anti-reflux surgery
Ureteral reimplantation in children with unrecognized dysfunctional voiding	Reflux secondary to bladder outlet dysfunction misdiagnosed as primary anatomical VUR	Postoperative persistence of reflux/incontinence; recurrent infections despite technically successful surgery	Treat functional outlet dysfunction (biofeedback, timed voiding, bowel management, antimuscarinics/α-blockers); reassess reflux after functional correction
Labeling incontinence or retention as behavioral without urodynamic confirmation	Lack of objective testing; underestimation of detrusor overactivity or underactivity	Delayed diagnosis; progression to hydronephrosis or renal scarring	Early non-invasive uroflow/PVR; cystometry when indicated; phenotype-guided therapy
Neglecting constipation or bowel dysfunction in LUTD management	Overlooking bladder–bowel interaction	Treatment failure; recurrent UTIs and incontinence	Integrated bowel regimen as part of standard urotherapy; dietary fiber, laxatives, timed toileting
Prolonged antibiotic prophylaxis without addressing voiding dysfunction	Treating infection consequence rather than the cause	Persistent bacteriuria and antimicrobial resistance	Functional evaluation and correction; prophylaxis only as temporary adjunct during therapy initiation
Proceeding to invasive or surgical intervention before multidisciplinary review	Fragmented care, absence of urodynamic input	Iatrogenic morbidity, continued symptoms	Multidisciplinary team evaluation (urology, nephrology, physiotherapy, psychology); individualized stepwise management

* Key message: A “function-first” diagnostic approach—combining detailed history, bladder diary, non-invasive urodynamics, and bowel assessment—prevents unnecessary surgical procedures and ensures durable symptom resolution.

## Data Availability

De-identified data are available from the corresponding author on reasonable request; public sharing is restricted by patient privacy and institutional policy.

## Supplementary Materials

The following supporting information can be downloaded at: https://www.mdpi.com/article/10.3390/pediatric18010020/s1, Supplementary Table S1: Baseline and follow-up urodynamic findings in three children with severe functional lower urinary tract dysfunction.; Supplementary Table S2: Follow-up outcomes and treatment adherence in three children with severe functional lower urinary tract dysfunction.

## Abbreviations

The following abbreviations are used in this manuscript:

LUTD	Lower Urinary Tract Dysfunction
UTI	Urinary Tract Infection
VCUG	Voiding Cystourethrography
ED	Emergency Department
