# Supplementary material for: Severe Lower Urinary Tract Dysfunction in Otherwise Healthy Children: A Three-Case Series and Narrative Review
Source: Pediatr Rep. 2026 Feb 3;18(1):20. doi: 10.3390/pediatric18010020 (PMC12921987; doi:10.3390/pediatric18010020)
Supplement: Supplementary file 1 [file pediatrrep-18-00020-s001.zip › Supplementary Table S1.pdf]

Supplementary Table S1: Baseline and follow-up urodynamic findings in three children with severe functional lower urinary tract dysfunction.

**Parameter**	**Case 1 – Detrusor Overactivity**	**Case 2 – Hinman Syndrome (DSD, Poor Compliance)**	**Case 3 – Detrusor Underactivity**
Age / Sex	7 y / Male	3 y / Female	10 y / Male
Baseline bladder capacity	Reduced (≈60% of expected for age)	Normal–slightly reduced	Increased (>130% of expected for age)
Compliance	Decreased (≈8–10 mL/cm H_2O)	Markedly decreased (<10 mL/cm H_2O)	Normal
Filling-phase detrusor activity	Marked overactivity with early uninhibited contractions	Early overactivity with poor compliance	Areflexic (no contractions during filling)
Voiding-phase pattern	Coordinated detrusor contraction, complete emptying	Detrusor–sphincter dyssynergia (simultaneous contraction)	Weak detrusor contraction, poor flow
Maximum detrusor pressure (Pdet max)	~40 cm H_2O	>40 cm H_2O during filling and voiding	<20 cm H_2O
Qmax (max flow rate)	10–12 mL/s	5 mL/s, staccato pattern	4 mL/s, weak stream
Post-void residual (PVR)	Minimal (<10% of capacity)	High (≈120 mL; ~40% of capacity)	High (>50% of capacity)
Associated findings	Trabeculated mucosa, pseudopolyps on cystoscopy	Severe trabeculation, VUR grade V, unilateral renal scarring	Bladder wall thickening, grade I VUR
Therapy initiated	Urotherapy + trospium + biofeedback	CAP + trospium + CIC + biofeedback	Tamsulosin + biofeedback
Follow-up bladder function (24–36 mo)	Normalized capacity and compliance; symptom-free except relapse after withdrawal	Normal voiding intervals, no residuals or febrile UTIs	Normalized emptying; no residual or retention episodes
Follow-up PVR	<10% of capacity	<10% of capacity	0–10 mL
Outcome summary	Functional recovery; mild relapse after discontinuation	Full functional and renal stabilization	Sustained recovery and continence

** Numerical values are approximate estimates derived from qualitative urodynamic descriptions in the clinical records; complete tracings were not available for all cases.*
