# Supplementary material for: Severe Lower Urinary Tract Dysfunction in Otherwise Healthy Children: A Three-Case Series and Narrative Review
Source: Pediatr Rep. 2026 Feb 3;18(1):20. doi: 10.3390/pediatric18010020 (PMC12921987; doi:10.3390/pediatric18010020)
Supplement: Supplementary file 1 [file pediatrrep-18-00020-s001.zip › Supplementary Table S2.pdf]

Supplementary Table S2. Follow-up outcomes and treatment adherence in three children with severe functional lower urinary tract dysfunction

| Parameter | Case 1 – Detrusor Overactivity | Case 2 – Hinman Syndrome (DSD, Poor Compliance) | Case 3 – Detrusor Underactivity |
|---|---|---|---|
| Follow-up duration | 30 months | 36 months | 24 months |
| Follow-up schedule | 3, 6, 12 months, then annually | 3, 6, 12 months, then annually | 3, 6, 12 months, then annually |
| UTIs after initiation of therapy | None while adherent; 1 febrile UTI after treatment withdrawal | No febrile or culture-proven UTIs | No UTIs reported |
| Continence / retention outcome | Full daytime continence; relapse of enuresis after treatment withdrawal, resolved after retreatment | Normal voiding intervals; continence achieved | Complete resolution of acute urinary retention episodes |
| Post-void residual (PVR) at last follow-up | <10% of bladder capacity | <10% of bladder capacity | 0–10 mL |
| Urodynamic evolution | Increased bladder capacity; suppression of detrusor overactivity | Improved compliance; resolution of dyssynergia and high-pressure voiding | Improved detrusor emptying; low residual volumes |
| Renal / upper tract outcome | Stable renal ultrasound; no reflux | Stable renal function; no new scarring on follow-up DMSA | Normalized bladder wall thickness on ultrasound |
| Adherence to conservative therapy | Good initially; interruption due to treatment withdrawal associated with relapse | Good adherence to CIC, CAP, and biofeedback during early management | Good adherence to biofeedback and pharmacologic therapy |
| Overall outcome | Functional improvement with relapse after discontinuation, responsive to retreatment | Sustained functional and renal stabilization | Sustained recovery and continence |

*Adherence was assessed based on parental report, physiotherapy/biofeedback session attendance, and clinical correlation with symptom recurrence or resolution. Follow-up urodynamic trends are detailed in Supplementary Table S1.*
